# Inhibition of *Plasmodium* Hepatic Infection by Antiretroviral Compounds

**DOI:** 10.3389/fcimb.2017.00329

**Published:** 2017-07-19

**Authors:** Marta Machado, Margarida Sanches-Vaz, João P. Cruz, António M. Mendes, Miguel Prudêncio

**Affiliations:** ^1^Faculdade de Medicina, Instituto de Medicina Molecular, Universidade de Lisboa Lisboa, Portugal; ^2^iMed.UL-Research Institute for Medicines and Pharmaceutical Sciences, Faculdade de Farmácia da Universidade de Lisboa Lisboa, Portugal

**Keywords:** *Plasmodium*, liver stage, malaria, HIV, AIDS, antiretrovirals, antiretroviral therapy

## Abstract

Recent WHO guidelines on control of human immunodeficiency virus (HIV) call for the widespread use of antiretroviral (AR) therapy (ART) for people living with HIV. Given the considerable overlap between infections by HIV and *Plasmodium*, the causative agent of malaria, it is important to understand the impact of AR compounds and ART regimens on infections by malaria parasites. We undertook a systematic approach to identify AR drugs and ART drug combinations with inhibitory activity against the obligatory hepatic stage of *Plasmodium* infection. Our *in vitro* screen of a wide array of AR drugs identified the non-nucleoside reverse transcriptase inhibitors efavirenz and etravirine (ETV), and the protease inhibitor nelfinavir, as compounds that significantly impair the development of the rodent malaria parasite *P. berghei* in an hepatoma cell line. Furthermore, we show that WHO-recommended ART drug combinations currently employed in the field strongly inhibit *Plasmodium* liver infection in mice, an effect that may be significantly enhanced by the inclusion of ETV in the treatment. Our observations are the first report of ETV as an anti-Plasmodial drug, paving the way for further evaluation and potential use of ETV-containing ARTs in regions of geographical overlap between HIV and *Plasmodium* infections.

## Introduction

The most recent WHO guidelines for treatment and prevention of infection with human immunodeficiency virus (HIV), the causative agent of acquired immunodeficiency syndrome (AIDS), recommend that all people living with HIV should be provided with antiretroviral (AR) therapy (ART), toward achieving universal access to HIV treatment and care, and ending AIDS as a public health threat (WHO, 2016a,b). These guidelines state that all populations and age groups are eligible for HIV treatment, including pregnant women and children, as well as adults living with HIV, including those with tuberculosis, hepatitis, and other co-infections (WHO, 2016a,b).

There is considerable geographic overlap between HIV and *Plasmodium*, the causative agent of malaria, particularly in sub-Saharan Africa, where factors such as limited access to healthcare facilities and widespread poverty favor the extensive transmission of either pathogen (Njunda et al., 2016). Thus, co-infection with *Plasmodium* and HIV is common in that region, contributing to the spread of both diseases, which remain formidable public health problems (Abu-Raddad et al., 2006; Skinner-Adams et al., 2008). Numerous reports suggest an altered clinical outcome for *Plasmodium*/HIV co-infected patients, with both infections enhancing each other's severity. In fact, HIV has been shown to increase the risk of development of severe *P. falciparum* malaria (Grimwade et al., 2004; Cohen et al., 2005; Patnaik et al., 2005; Otieno et al., 2006; Flateau et al., 2011), while malaria has been associated with a decline in CD4^+^ T cell counts (Patnaik et al., 2005), enhanced HIV-1 replication (Kublin et al., 2005) and increased HIV transmission (Abu-Raddad et al., 2006). In light of these observations, WHO's “ART-for-all” recommendation warrants an in-depth understanding of HIV drug influence on *Plasmodium* infection. However, while several reports have focused on the impact of ART on clinical malaria (reviewed in Van Geertruyden, 2014; Hobbs and Parikh, 2017) and on pharmacokinetic interactions between AR and antimalarial drugs (reviewed in Fehintola et al., 2011; Van Geertruyden, 2014), fewer studies exist on the specific impact of AR drugs on the clinically silent, yet statutory hepatic stage of infection by *Plasmodium* parasites.

The hepatic stage of *Plasmodium* infection is the initial stage of mammalian infection by malaria parasites. Sporozoites injected through the bite of infected female *Anopheles* mosquitoes travel to their host's liver, where they invade hepatocytes. Following invasion, sporozoites differentiate into exoerythrocytic forms (EEFs) that undergo a period of extensive replication, termed development. Hepatic infection culminates in the release of several thousand red blood cell (RBC)-infective merozoites into the bloodstream, where they cyclically infect RBCs, giving rise to malaria symptoms and originating gametocytes that warrant the progress of infection onto the mosquito vector (Prudencio et al., 2006). Current tools for malaria control are precarious and recent calls have been made for developing new or repurposing existing drugs as valuable interventions to help control infection (Alonso et al., 2011). The asymptomatic but obligatory nature of the hepatic stage of *Plasmodium* infection makes it a privileged target for anti-Plasmodial intervention, as drugs capable of inhibiting the parasite's liver stages (LS) could effectively impair infection before the onset of disease (Prudencio et al., 2006; Derbyshire et al., 2012; Rodrigues et al., 2012). Moreover, certain *Plasmodium* species, such as *P. vivax* and *P. ovale*, can produce chronic liver forms termed hypnozoites, which may remain dormant for extended periods of time before relapsing. Primaquine (PQ), currently the only licensed drug that can clear hypnozoites to achieve radical cure of infections by *P. vivax* and *P. ovale*, presents potentially lethal side effects (Baird and Hoffman, 2004; Vale et al., 2009).

The few existing studies on the impact of AR drugs on the hepatic stages of malaria parasites are suggestive of their potential impact on *Plasmodium* liver infection. *In vitro* studies showed that saquinavir (SQV), ritonavir (RTV), indinavir (IDV) and lopinavir (LPV) displayed activity against the hepatic stages of rodent *P. yoelii* (Mahmoudi et al., 2008) and/or *P. berghei* (Hobbs et al., 2009) parasites, whereas LPV, SQV, and nevirapine (NVP) were active against the human-infective *P. falciparum* (Hobbs et al., 2013a). *In vivo*, a modest reduction of *P. yoelii* liver stage burden was observed following treatment by the non-nucleoside reverse transcriptase inhibitors (NNRTIs) efavirenz (EFV), etravirine (ETV) and NVP (Hobbs et al., 2012), whereas LPV+RTV displayed a dose-dependent effect on this parasite's hepatic development (Hobbs et al., 2009). However, a more recent study showed that the latter drug combination inhibited *P. knowlesi* pre-erythrocytic stages in *Rhesus* monkeys only when provided in combination with the antibiotic trimethoprim sulfamethoxazole (TMP-SMX) (Hobbs et al., 2014).

In the present study, we employed a variety of well-established methods to assess the effect of a wide range of HIV inhibitors, belonging to the main classes of AR compounds, on hepatic infection by the *P. berghei* rodent malaria parasite, a commonly employed and widely accepted model for *Plasmodium* infection studies (Prudencio et al., 2011). We identified several compounds that potently inhibit *P. berghei* LS *in vitro* and *in vivo* through a strong reduction of the numbers of LS parasites and a clear impairment of their ability to develop inside hepatic cells. We further evaluated the impact of currently recommended ART regimens, as well as of alternative drug combinations, on liver infection by malaria parasites. The knowledge generated by our study provides important information that can help guide ART strategies in the context of areas of *Plasmodium* and HIV co- endemicity.

## Results

### *In vitro* screening of antiretroviral compounds active against *Plasmodium* hepatic stages

We initiated our study by evaluating the *in vitro* activity of members of the 4 main classes of AR compounds, nucleoside reverse transcriptase inhibitors (NRTIs), NNRTIs, protease inhibitors (PIs) and integrase inhibitors (II) (Table 1), against *P. berghei* infection of hepatic cells. To this end, we initially made use of an *in vitro* infection model that employs a luciferase-expressing *P. berghei* (*Pb*_Luc_) parasite line, whose infectivity is indistinguishable from that of wild-type *P. berghei*, and a human hepatoma cell line, HuH7 (Ploemen et al., 2009). HuH7 cells were infected with *Pb*_Luc_ sporozoites and parasite loads were assessed 48 h post-infection (hpi) by bioluminescence measurements of cell lysates. The IC50 and IC90 concentrations of each compound under evaluation were determined by assessing infection loads following incubation with various concentrations of each compound (Table 1). Our results show that NNRTIs and PIs are the classes of AR compounds most active against *Plasmodium* hepatic infection, whereas no substantial activity was observed for the NRTIs nor the II tested. Three out of 4 NNRTIs (ETV, EFV and rilvipirine-RIL) and 8 out of 9 PIs (LPV, RTV, SQV, nelfinavir-NFV, atazanavir-ATV, telapravir-TLV, darunavir-DRV and amprenavir-APV) evaluated in our assay displayed marked activity against *P. berghei* hepatic stages, with IC50s ranging from ~2.7 to ~23.4 μM (Table 1). Importantly, CellTiter-based measurements of cell proliferation showed that none of the compounds showed any toxicity against the host cells in the concentrations employed in the study, therefore displaying selectivity for parasites over the mammalian cell line tested (Table 1).

**Table 1:** Activity of antiretroviral drugs on overall *P. berghei in vitro* hepatic infection.

**Class**	**Drug**	**IC50 (μM) ± *SD***	**IC90 (μM) ± *SD***	Cell proliferation^**^ (%) ± *SD*
Protease inhibitors (PIs)	Nelfinavir (NFV)	2.7 ± 0.6	10.9 ± 3.1	110.2 ± 6.7
	Lopinavir (LPV)	3.9 ± 0.9	11.1 ± 2.1	110.5 ± 18.2
	Ritonavir (RTV)	6.8 ± 1.4	22.2 ± 2.6	93.5 ± 3.6
	Saquinavir (SQV)	8.0 ± 1.7	13.1 ± 2.7	114.4 ± 2.8
	Atazanavir (ATV)	10.6 ± 0.8	27.0 ± 4.8	105.3 ± 3.1
	Telapravir (TLV)^*^	13.3 ± 1.7	23.9 ± 5.5	114.2 ± 13.9
	Darunavir (DRV)	16.8 ± 2.8	44.1 ± 8.6	118.7 ± 10.1
	Amprenavir (APV)	23.4 ± 5.1	70.9 ± 9.2	100.1 ± 4.8
	Indinavir (IDV)	>50	>50	102.9 ± 4.6
Non-nucleoside reverse transcriptase inhibitors (NNRTIs)	Rilvipirine (RIL)	4.3 ± 1.1	10.2 ± 2.3	99.4 ± 12.0
	Etravirine (ETV)	4.4 ± 0.7	10.5 ± 1.8	94.4 ± 8.3
	Efavirenz (EFV)	17.2 ± 1.8	23.9 ± 3.7	111.2 ± 13.7
	Nevirapine (NVP)	>50	>50	103.4 ± 8.6
Nucleoside reverse transcriptase inhibitors (NRTIs)	Abacavir (ABC)	>50	>50	102.7 ± 10.6
	Emtricitabine (FTC)	>50	>50	104.8 ± 12.8
	Lamivudine (3TC)	>50	>50	106.4 ± 10.8
	Tenofovir (TDF)	>50	>50	104.7 ± 6.1
	Zidovudine (AZT)	>50	>50	100.6 ± 11.9
Integrase inhibitor (II)	Raltegravir (RAL)	>50	>50	120.9 ± 16.7

*IC50 and IC90, concentrations inhibiting 50 and 90% of in vitro infection, respectively; SD, standard deviation*.

* *Treatment of Hepatitis C*.

** Measured by CellTiter assay at ~IC90 drug concentrations, relative to the DMSO control

### Compound effect on the establishment of infection and on intra-hepatic parasite development

**Figure 1:**
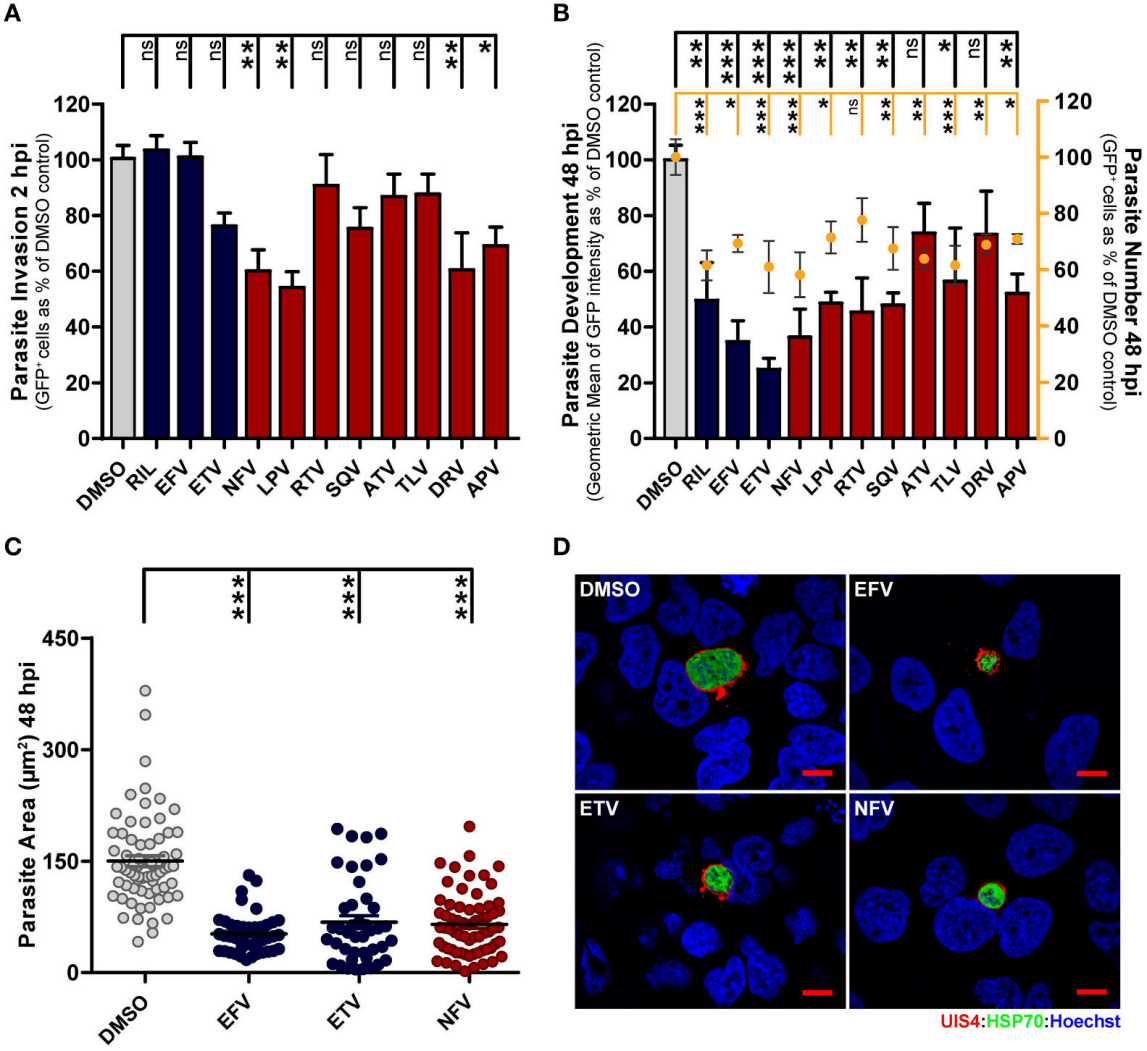
Antiretroviral drugs inhibit *Plasmodium* hepatic infection *in vitro*. **(A,B)** HuH7 cells were infected with green fluorescent protein (GFP)-expressing *P. berghei* sporozoites. The culture medium of HuH7 cells was replaced by medium containing the IC50 concentration of selected drugs 1 hour prior to infection or 2 hpi, for invasion and development assays, respectively. **(A)** Cell invasion was quantified by flow cytometry by determining the percentage of GFP^+^ cells at 2 hpi. **(B)** Percentage of infected cells (dots) and parasite development (bars) were assessed by determining the percentage and fluorescence intensity of GFP^+^ cells at 48 hpi, respectively. **(C)** Parasite development in cells treated with EFV, ETV and NFV was also assessed by quantification of EEF area by immunofluorescence microscopy. **(D)** Representative immunofluorescence microscopy images show *P. berghei* hepatic forms treated with the IC50 concentrations of EFV, ETV and NFV or with DMSO (control) from 2 to 48 hpi. Immunofluorescence microcopy employed antibodies against *Pb*UIS4 (a parasitophorous vacuole membrane (PVM) protein, in red), *Pb*HSP70 (a heat shock protein that localizes to the parasite soma, in green), and the nuclear stain Hoechst (in blue). Scale bars, 10 μm. Plots represent the mean values of at least three independent experiments with error bars indicating SEM. One-way ANOVA with post-test Dunnett. ns, not significant, ^*^ *p* < 0.05, ^**^ *p* < 0.01, ^***^ *p* <0.001. Light gray bars or circles in panels **(A–C)** correspond to solvent controls, blue bars or circles correspond to NNRTIs, and red bars or circles correspond to PIs.

During infection of hepatic cells by *Plasmodium*, a cell invasion stage, completed within up to 2 h after sporozoite addition, is followed by a parasite development period, lasting ~60 h *in vitro*. Thus, having identified the overall most active AR compounds in our luminescence-based drug screen, we next used a flow cytometry-based methodology to independently assess these compound's ability to block the establishment of infection and/or to impair parasite development in the hepatic cells (Prudencio et al., 2008). Flow cytometry analysis of GFP-expressing *P. berghei* (*Pb*_*GFP*_)-infected cells 2 h after sporozoite addition and incubation with the previously determined IC50 concentrations of each selected compound showed that NFV, LPV, DRV, and APV significantly impair cell invasion by the parasites, indicating that these compounds can inhibit the onset of infection (Figure 1A). Our results also show that most compounds decrease the percentage of GFP-positive cells 48 h after infection, suggesting an elimination of infected cells throughout that period (Figure 1B). An analysis of GFP intensity of infected cells treated for 48 h with IC50 concentrations of the drugs further revealed that all the compounds under study inhibit parasite development, with EFV, ETV, and NFV leading to the strongest inhibition (65.3, 75.2, and 63.6%, respectively) of parasite growth (Figure 1B). These results were further supported by a subsequent immunofluorescence microscopy analysis of *Pb*_Luc_-infected cells 48 h after sporozoite addition and incubation with the IC50 concentrations of EFV, ETV, and NFV, which unequivocally confirmed that these compounds markedly impair parasite development inside their hepatic host cells (Figures 1C,D).

Collectively, our data show that several commonly used AR drugs are active against *Plasmodium* LS *in vitro*, inhibiting *P. berghei* replication inside hepatic cells and, in some cases, impairing the establishment of infection.

### Inhibition of *Plasmodium* liver infection *in vivo* by antiretroviral compounds

**Figure 2:**
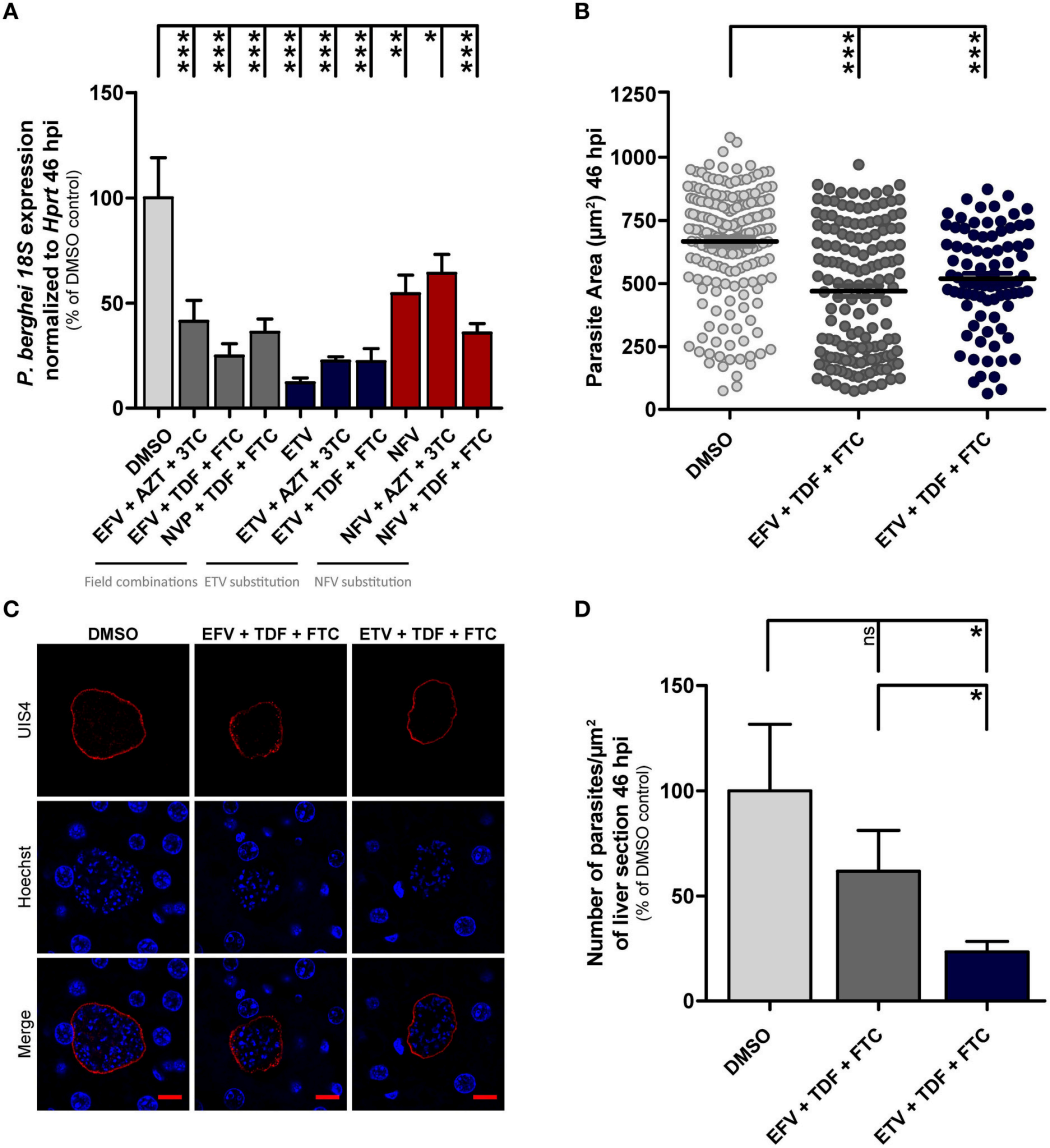
Antiretroviral drugs decrease liver *Plasmodium* infection *in vivo*. **(A)** Parasite infection loads measured by qRT-PCR in the livers of mice treated with the various compounds, relative to those of DMSO-treated controls, at 46 hpi. **(B)** Effect of drug treatment on parasite size as a surrogate of parasite development at 46 hpi. Analysis was performed by immunofluorescence microscopy through quantification of EEF area. **(C)** Representative confocal microscopy images of liver parasites in treated and control mice. Red, *Pb*UIS4 labeling showing the PVM; blue, Hoechst nuclear staining. Scale bars, 10 μm. **(D)** Parasite density per square millimeter of mouse liver sections following treatment with selected drug combinations, or with the vehicle, at 46 hpi. Plots represent the mean values of at least two independent experiments with error bars indicating SEM. One-way ANOVA with post-test Dunnett. ns, Not significant, ^*^ *p* < 0.05, ^**^ *p* < 0.01, ^***^ *p* < 0.001. Light gray bars or circles in panels **(A,B,D)** correspond to vehicle controls, dark gray bars or circles correspond to field combinations, blue bars or circles correspond to replacement with the NNRTI ETV, and red bars in **(A)** correspond to replacement with the PI NFV.

Having established the effect of a variety of individual AR compounds on *Plasmodium* hepatic infection *in vitro*, we sought to analyze how their proposed use in accordance with WHO guidelines for ART could impact *Plasmodium* infection *in vivo*. These guidelines recommend that first-line ART for adults and adolescents should consist of two NRTIs plus a NNRTI or an II. EFV+Tenofovir (TDF) + lamivudine (3TC) (or emtricitabine—FTC) as a fixed-dose combination is recommended as the preferred option to initiate ART. Recommended alternatives include EFV+3TC+zidovudine (AZT), NVP+AZT+3TC and NVP+TDF+3TC (or FTC). For children 3 to 10 years of age, the NRTI backbone should be either abacavir (ABC)+3TC or TDF+3TC (or FTC). For children 3 years and older, EFV is the preferred NNRTI for first-line treatment and NVP is the preferred alternative (WHO, 2016a). Thus, we decided to evaluate the impact of the recommended field combinations EFV+AZT+3TC, EFV+TDF+FTC, and NVP+TDF+FTC on *Plasmodium* hepatic infection. Additionally, we further assessed the *in vivo* efficacy of alternative drugs or drug combinations, based on the results of our *in vitro* screening analysis, which identified EFV and ETV as the NNRTIs, and NFV as the PI with the strongest impact on *Plasmodium* development. Thus, we evaluated the effect of ETV and NFV alone, as well as of combinations where the recommended EFV and NVP NNRTIs were replaced by either ETV or NFV (ETV+AZT+3TC, ETV+TDF+FTC, NFV+AZT+3TC, and NFV+TDF+FTC). In these experiments, mice were infected with *Pb*_Luc_ sporozoites, and treated with allometry-scaled doses of the different drugs or drug combinations, administered according to the recommended schedule (Supplementary Figure 1). Liver parasite loads were determined 46 h after infection and compared to those of untreated controls (Figure 2A). Our results show that all the WHO-recommended drug combinations for HIV treatment tested in our study significantly decrease *Plasmodium* liver infection. Moreover, the NNRTI ETV alone or in any of the combinations employed inhibits *Plasmodium* infection to an either similar or larger extent than the WHO-recommended formulations. To further dissect the *in vivo* anti-Plasmodial effect of the first-line HIV treatment (EFV+TDF+FTC) and of the alternative combination (ETV+TDF+FTC), we analyzed liver sections of infected mice treated with either drug combination by immunofluorescence microscopy. Our results show that, while both combinations significantly decrease hepatic parasite development (Figures 2B,C), the latter also leads to a significant decrease in the number of EEFs developing inside hepatocytes (Figure 2D).

Overall, our results show that the ARTs currently employed in HIV treatment have a significant impact on liver infection by *Plasmodium* parasites, an effect that may be further enhanced by replacing EFV by the alternative NNRTI ETV.

## Discussion

Repurposing strategies for drug discovery in the field of infectious diseases have emerged as an attractive fast-track approach to speed up the development of drugs against these diseases (Kaiser et al., 2015; Klug et al., 2016). Given their extensive characterization over decades of development (Pau and George, 2014), AR drugs constitute an appealing class of compounds for such repurposing approaches, particularly those aimed at controlling malaria. Most importantly, understanding the effect of AR compounds and ART on *Plasmodium* infection is of great clinical relevance, particularly in regions of HIV/*Plasmodium* co-endemicity. While most studies on this subject have concentrated on evaluating the impact of AR compounds on the blood stages of human- (Skinner-Adams et al., 2004; Andrews et al., 2006; Redmond et al., 2007; Lek-Uthai et al., 2008; Nsanzabana and Rosenthal, 2011) and rodent-infective (Andrews et al., 2006; Martins et al., 2006; Akinyede et al., 2013; Abiodun et al., 2015) malaria parasites (Hobbs et al., 2013b), much less is known on their effect on the pre-erythrocytic stage of *Plasmodium* infection.

In the present report, we undertook a systematic approach to evaluate the anti-Plasmodial effect of an array of 19 AR compounds, belonging to 4 distinct classes of AR drugs, with the sole focus on their activity against the hepatic stage of infection by the malaria parasite. Our *in vitro* results showed that the NNRTIs EFV, and ETV, and the PI NFV exert a strong anti-Plasmodial activity through a marked impairment of the parasite's ability to develop inside its host cells. Given the geographical overlap between HIV and *Plasmodium* infections, it is extremely likely that patients undergoing ART may become infected by malaria parasites. As such, we further evaluated the effect of the WHO-recommended ART drug combinations (WHO, 2016a) on *in vivo* infection by *Plasmodium* liver stages in HIV-free mice. Our results showed that all the three field combinations employed in our assay displayed strong inhibitory activity against *P. berghei* liver infection. Of note, EFV was the only individual component in these combinations that showed anti-Plasmodial activity *in vitro*, suggesting a possible synergy between the different components of the combination *in vivo*. Interestingly, synergistic effects have been reported for FTC and TDF in terms of anti-HIV-1 activity (Kulkarni et al., 2014), in agreement with the notion that a combination of different agents provides synergistic or additive antiviral effects (Pau and George, 2014).

Additionally, we assessed the effect of alternative drug combinations where the NRTI components of the mixture were maintained but the NNRTIs EFV or NVP were replaced by either the NNRTI ETV or the PI NFV, identified in our screen. This analysis showed that whereas replacing the NNRTIs present in the original mixture by NFV had no positive impact on the inhibition of *Plasmodium* liver infection, their replacement by ETV led not only to an equivalent deficiency in parasite growth but also to an additional significant decrease in the number of parasite EEFs developing inside liver cells. Importantly, while both EFV and ETV have been shown to inhibit erythrocytic infection by *P. falciparum in vitro*, the reported IC50 for ETV is approximately 10-fold lower than for EFV (Nsanzabana and Rosenthal, 2011). Also of note, EFV has been shown to decrease the plasma concentration of atovaquone/proguanil, a drug combination frequently used in malaria prophylaxis, potentially leading to failures thereof (van Luin et al., 2010). Interestingly, atovaquone/proguanil prophylaxis leads to an increase in ETV plasma concentration (Tommasi et al., 2011), which might prove beneficial when ETV-based ARV treatment is administered in the context of malaria prophylaxis. Finally, while only minimal hepatotoxicity has been observed for ETV (Johnson and Saravolatz, 2009), incubation of human liver cells with EFV results in marked oxidative stress and apoptosis (Apostolova et al., 2010). Although it has been speculated that reactive oxygen species may in fact be involved in EFV-induced parasite toxicity (Hobbs et al., 2012), the anti-Plasmodial mechanism of action of NNRTIs remains hitherto unknown.

Our study constitutes the first reported evidence of a strong inhibitory effect of the NNRTI ETV on *Plasmodium* hepatic stages, either *in vitro* or *in vivo*, and when administered to mice as a single drug or in combination with other compounds. Curiously, lower incidence of malaria has been described for HIV-infected children receiving ARTs based on the PIs LPV+RTV, compared with those receiving NNRTI-based therapy (Achan et al., 2012). Very recently, evidence was also found that PI-containing ART regimens may be associated with a lower clinical malaria incidence compared with NRTI only or NNRTI-containing regimens (Kasirye et al., 2017). However, the NNRTIs employed for ART in these studies were either EFV or NVP (Achan et al., 2012; Ugandan Ministry of Health, 2013), and no reports exist on the clinical impact of ETV on malaria. Thus, our observations warrant a more detailed evaluation of the anti-Plasmodial potential of ETV and of the employment of ETV-containing ARTs in regions of geographical overlap between HIV and *Plasmodium* infections.

## Materials and methods

### Drugs and chemicals

Antiretroviral drugs were obtained from the NIH AIDS Research and Reference Reagent Program. For the *in vitro* studies, compounds were dissolved in dimethyl sulfoxide (DMSO). For the *in vivo* studies, compounds were first dissolved in DMSO and further diluted in sunflower oil for oral administration. RPMI 1640, PBS pH 7.4, trypsin, fetal bovine serum (FBS), non-essential amino acids, penicillin/streptomycin, glutamine and HEPES pH 7 were purchased from Gibco/Invitrogen. All other chemicals were obtained from Sigma, unless otherwise specified.

### Mice, cells and parasites

Male C57Bl/6J mice (Charles River laboratories), 6–8 weeks of age, were used. Mice were housed, kept under specific pathogen-free (SPF) conditions and manipulated in the animal facility of the Instituto de Medicina Molecular (Lisbon, Portugal). All animal experiments were performed in strict compliance to the guidelines of our institution's animal ethics committee, who also approved the study, and the Federation of European Laboratory Animal Science Associations (FELASA). HuH7 cells, a human hepatoma cell line, were cultured in RPMI 1640 medium supplemented with 10% FBS (v/v), 0.1 mM non-essential amino acids, 50 μg/mL penicillin/streptomycin, 2 mM glutamine and 1 mM HEPES (final concentrations), pH 7 and maintained at 37°C with 5% CO_2_. Fungizone and gentamycin were added at 1:500 and 1:1,000, respectively. Green Fluorescent Protein (GFP)- or luciferase-expressing *P. berghei* ANKA sporozoites were freshly isolated from infected *Anopheles stephensi* mosquitoes, reared at Instituto de Medicina Molecular, prior to being employed for *in vitro* and *in vivo* infections.

### Quantification of overall *in vitro* infection by luminescence

Overall hepatic infection was determined by measuring the luminescence intensity of lysates of HuH7 cells infected with a firefly luciferase-expressing *P. berghei* line, as previously described (Ploemen et al., 2009). Briefly, HuH7 cells (1.0 × 10^4^ per well) were seeded in 96-well plates the day before infection. The medium was replaced approximately 1 h prior to infection by the appropriate drug- or vehicle-containing medium. In control wells, the compound's vehicle (DMSO) was added in an amount equivalent to that present in the highest drug concentration employed. Sporozoite addition (1.0 × 10^4^ per well) was followed by centrifugation at 1,800 × g for 5 min. Parasite infection load was measured 48 hpi by a bioluminescence assay (Biotium) using a multi-plate reader Infinite M200 (Tecan). The effect of the different treatments on the viability of HuH7 cells was assessed by the CellTiter-Blue assay (Promega) according to the manufacturer's protocol. Non-linear regression analysis was employed to fit the normalized results of the dose-response curves, and IC50 and IC90 values were determined using GraphPad Prism V 5.0.

### Quantification of *P. berghei* invasion and development by flow cytometry

Invasion of hepatoma cells and development of intracellular parasites were assessed by determining the percentage of GFP^+^ cells 2 hpi with a GFP-expressing *P. berghei* line and by measuring the intensity of the GFP signal of the infected cells 48 hpi, respectively, as previously described (Prudencio et al., 2008). HuH7 cells (1.0 × 10^4^ per well) were seeded in 96-well plates the day before infection. The medium was replaced by the appropriate drug- or vehicle-containing medium 1 h prior or 2 hpi, for invasion and development quantification, respectively. In control wells, the compound's vehicle (DMSO) was added in an amount equivalent to that present in the highest drug concentration employed. Sporozoite addition (1.0 × 10^4^ per well) was followed by centrifugation at 1,800 × g for 5 min. Cells were then collected for flow cytometry analysis at 2 or 48 hpi, respectively, and analyzed on a BD LSR Fortessa flow cytometer with the DIVA software (version 6.2). Analysis was carried out using the FlowJo software (version 6.4.7, FlowJo).

### Immunofluorescence microscopy analysis of *in vitro* and *in vivo* hepatic infection

For *in vitro* immunofluorescence microscopy analyses, 5.0 × 10^4^ cells were seeded on glass coverslips in 24-well plates. The medium was replaced approximately 2 h prior to infection by the appropriate drug- or vehicle-containing medium. In control wells, the compound's vehicle (DMSO) was added in an amount equivalent to that present in the highest drug concentration employed. Sporozoite addition (2.0 × 10^4^ per well) was followed by centrifugation at 1,800 × g for 5 min. Forty-eight hour after infection, cells were rinsed with PBS, fixed with 4% v/v paraformaldehyde (PFA; Santa Cruz Biotechnology) for 20 min at room temperature, washed 3 times with PBS, and stored at 4°C. Cells were incubated with permeabilization/blocking solution (0.1% v/v Triton X-100, 1% w/v bovine serum albumin (BSA) in 1 × PBS) for 30 min at room temperature. Parasites were stained with a mouse monoclonal antibody against the parasite-specific Heat Shock Protein 70 (Hsp70) (2E6; dilution 1:100) and a goat anti-UIS4 antibody (dilution 1:1,000) for 1 h at room temperature and washed with PBS. Cells were further incubated in a 1:400 dilution of anti-mouse Alexa-Fluor 488 (Jackson ImmunoResearch Laboratories) or anti-goat Alexa-Fluor 568 (Life Technologies) secondary antibodies along with a 1:1,000 dilution of Hoechst 33342 (Invitrogen), for 30 min at room temperature, washed with PBS, and mounted in Fluoromount G (Southern Biotech). For *in vivo* studies, 50 micrometer sections of fixed livers collected at 46 hpi were similarly stained and analyzed as previously described (Meireles et al., 2017; Mendes et al., 2017). Confocal images were acquired using a Zeiss LSM 710 confocal microscope. Widefield images for size determination were acquired in a Zeiss Axiovert 200 M microscope. Images were processed with ImageJ software (version 1.47).

### Quantification of *in vivo* hepatic infection

C57Bl/6J mice were infected by intravenous injection of 2.0 × 10^4^ firefly luciferase–expressing *P. berghei* sporozoites. The compounds were administered in sunflower oil by oral gavage at specific schedules (Supplementary Figure 1). An equivalent amount of drug vehicle (DMSO) was administered in control mice. Liver parasite burden of infected mice was quantified by quantitative real-time PCR (qRT-PCR) as previously described (23). Briefly, livers were collected at 46 hpi and immediately homogenized in denaturing solution (4 M guanidine thiocyanate, 25 mM sodium citrate pH 7.0, 0.5% (w/v) sarcosyl and 0.7% (v/v) β-mercaptoethanol in DEPC-treated water). Total RNA was extracted using the TripleXtractor directRNA Kit (GRiSP), according to the manufacturer's protocol. One microgram of total RNA was converted to cDNA [NZY First-Strand cDNA Synthesis Kit, no oligonucleotides, (NZYTech)], and parasite load was quantified by qRT-PCR using primers specific for *P. berghei* 18S RNA (5′-AAGCATTAAATAAAGCGAATACATCCTTAC-3′ and 5′-GGAGATTGGTTTTGACGTTTATGTG-3′). Primers for the housekeeping gene hypoxanthine-guanine phosphoribosyltransferase *(Hprt)* (5′-TTTGCTGACCTGCTGGATTAC-3′ and 5′- CAAGACATTCTTTCCAGTTAAAGTTG -3′) were used for normalization of infection load in all experiments. The qRT-PCR reactions were performed in a total volume of 20 μL in an ABI Prism 7500 Fast system (Applied Biosystems) using the iTaqTM Universal SYBR® Green kit (BioRad) as follows: 50°C for 2 min, 95°C for 10 min, 40 cycles at 95°C for 15 s and 60°C for 1 min, melting stage was done at 95°C for 15 s, 60°C for 1 min, and 95°C for 30 s. The delta-delta cycle threshold (ΔΔCT) relative quantification method was used for analysis of qRT-PCR results.

### Statistical analyses

Statistically significant differences between control and treated conditions were analyzed using the One-way ANOVA with post-test Dunnett with a 95% confidence interval. Results were considered to be: ns, not significant, ^*^p < 0.05, ^**^p < 0.01, ^***^p < 0.001. All statistical tests were performed by GraphPad Prism V 5.0.

## Author contributions

MM and MS performed the experimental work and revised the manuscript. JC contributed reagents and provided intellectual input. AM provided intellectual input and contributed to writing the manuscript. MP supervised the work and wrote the manuscript.

### Conflict of interest statement

The authors declare that the research was conducted in the absence of any commercial or financial relationships that could be construed as a potential conflict of interest.

## Supplementary material

Supplementary Figure 1 *In vivo* experimental setup. **(A)** Schedules and doses of administration of antiretroviral drugs. **(B)** Schematic illustration of the *in vivo* experimental setup, highlighting the schedules of drug treatment employed.
