# Supplementary material for: Inhibition of Plasmodium Hepatic Infection by Antiretroviral Compounds
Source: Front Cell Infect Microbiol. 2017 Jul 19;7:329. doi: 10.3389/fcimb.2017.00329 (PMC5515864; doi:10.3389/fcimb.2017.00329)

**A**

| | | Dose in humans (w) | Dose in mice (w/W)* | Schedule |
|---|---|---|---|---|
| FIELD COMBINATION | EFV + AZT + 3TC | 600 mg EFV | 62.4 mg/kg EFV | 24/24h |
| | | 300 mg ZDV | 31.2 mg/kg ZDV | 12/12h |
| | | 150 mg 3TC | 15.6 mg/kg 3TC | 12/12h |
| | EFV + TDV + FTC | 600 mg EFV | 62.4 mg/kg EFV | 24/24h |
| | | 300 mg TDV | 31.2 mg/kg TDV | 24/24h |
| | | 200 mg FTC | 20.8 mg/kg FTC | 24/24h |
| | NVP + TDV + FTC | 200 mg NVP | 20.8 mg/kg FTC | 24/24h |
| | | 300 mg TDV | 31.2 mg/kg TDV | 24/24h |
| | | 200 mg FTC | 20.8 mg/kg FTC | 24/24h |
| ETV SUBSTITUTION | ETV | 200 mg ETV | 20.8 mg/kg ETV | 12/12h |
| | ETV + AZT + 3TC | 200 mg ETV | 20.8 mg/kg ETV | 12/12h |
| | | 300 mg ZDV | 31.2 mg/kg ZDV | 12/12h |
| | | 150 mg 3TC | 15.6 mg/kg 3TC | 12/12h |
| | ETV + TDV + FTC | 200 mg ETV | 20.8 mg/kg ETV | 12/12h |
| | | 300 mg TDV | 31.2 mg/kg TDV | 24/24h |
| | | 200 mg FTC | 20.8 mg/kg FTC | 24/24h |
| NFV SUBSTITUTION | NFV | 1250 mg NLF | 130.0 mg/kg NFV | 12/12h |
| | NFV + AZT + 3TC | 1250 mg NLF | 130.0 mg/kg NFV | 12/12h |
| | | 300 mg ZDV | 31.2 mg/kg ZDV | 12/12h |
| | | 150 mg 3TC | 15.6 mg/kg 3TC | 12/12h |
| | NFV + TDV + FTC | 1250 mg NLF | 130.0 mg/kg NFV | 12/12h |
| | | 300 mg TDV | 31.2 mg/kg TDV | 24/24h |
| | | 200 mg FTC | 20.8 mg/kg FTC | 24/24h |

*- Allometry-scaled dose

**B**

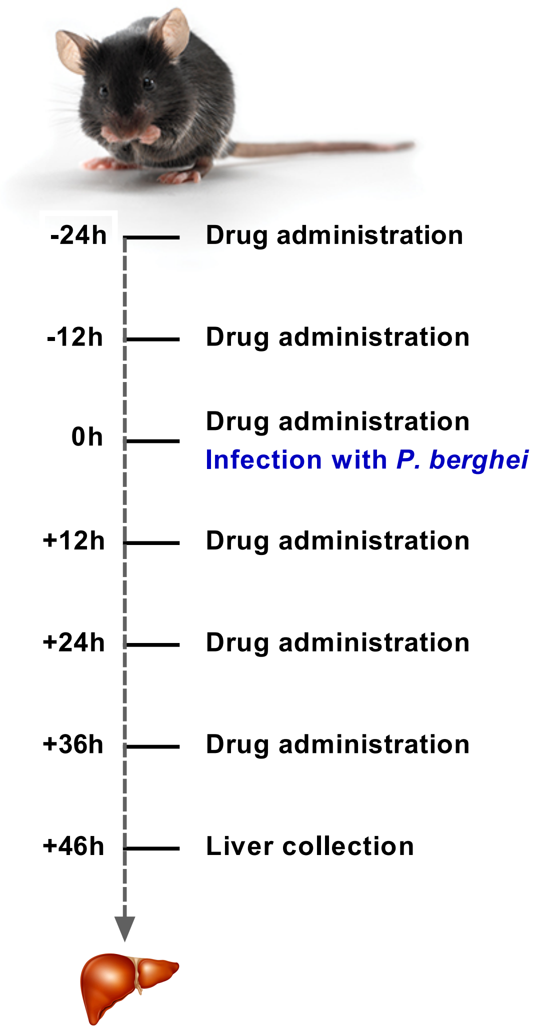

Supplement: Supplementary Figure 1 — In vivo experimental setup. (A) Schedules and doses of administration of antiretroviral drugs. (B) Schematic illustration of the in vivo experimental setup, highlighting the schedules of drug treatment employed. [file Image1.PDF]
